# Complete genome sequence of *Levilactobacillus acidifarinae* type strain JCM 15949 (DSM 19394)

**DOI:** 10.1128/mra.00374-25

**Published:** 2025-06-30

**Authors:** Mugihito Oshiro, Keisuke Nakamura, Rahul Sk, Yukihiro Tashiro, Yuh Shiwa

**Affiliations:** 1 Laboratory of Soil and Environmental Microbiology, Division of Systems Bioengineering, Department of Bioscience and Biotechnology, Faculty of Agriculture, Graduate School, Kyushu University 12923, Fukuoka, Fukuoka Prefecture, Japan; 2 NODAI Genome Research Center, Tokyo University of Agriculture 13126, Setagaya, Tokyo, Japan; 3 Department of Molecular Microbiology, Faculty of Life Sciences, Tokyo University of Agriculture 13126, Setagaya, Tokyo, Japan; The University of Arizona, Tucson, Arizona, USA

**Keywords:** lactic acid bacteria, sourdough, *Levilactobacillus*, Nanopore, long read, complete genome

## Abstract

Here, we report the complete genome sequence of *Levilactobacillus acidifarinae* type strain JCM 15949 (DSM 19394), which was isolated from a Belgian artisanal wheat sourdough. The genome consisted of a circular chromosome (2,915,962 bp, 51.71% GC content) and a circular plasmid (30,910 bp, 39.78% GC content).

## ANNOUNCEMENT

*Levilactobacillus* are free-living heterofermentative lactic acid bacteria (1). The genome sequence of the *Levilactobacillus acidifarinae* type strain isolated from a Belgian artisanal wheat sourdough (2) was reported using strain DSM 19394 (3) as a draft sequence. This study reports the complete genome sequence of *L. acidifarinae* type strain JCM 15949.

A frozen glycerol stock of the strain JCM 15949 was thawed and purified on a De Man–Rogosa–Sharpe (MRS) agar plate at 30°C. A single colony grown on the plate was picked up and inoculated to MRS broth. The broth cultured cells were harvested, washed, and suspended in TESS solution (25 mM Tris-HCl, 5 mM EDTA, 50 mM NaCl, 25% sucrose, pH 7.5). Genomic DNA was then extracted using a previously described method with some modifications (4–6). The cell suspension was incubated at 37°C with lysozyme and RNase. It was further incubated at 50°C with 10% sodium dodecyl sulfate and proteinase K. Genomic DNA was extracted using phenol-chloroform-isoamyl alcohol with 6 M NaCl, followed by purification with chloroform-isoamyl alcohol. Purified DNA was precipitated with 2-propanol at –20°C and then centrifuged. The resulting precipitate was rinsed with 90% ethanol, dried, and dissolved in Milli-Q water. The DNA was concentrated using a DNA Clean and Concentrator Kit (Zymo Research), which was quantified using a Qubit fluorometer. DNA quality was assessed using an Agilent TapeStation. A sequencing library was constructed using a Ligation Sequencing gDNA Native Barcode Kit 24 V14 (SQK-NBD114.24). Genomic DNA (1 µg) was sequenced on the FLO-MIN114 flow cell of a MinION Mk1B. Raw sequencing reads were obtained using MinKNOW v24.02.10, and bases were called using the super-accurate mode v5.0.0. Dorado v0.9.1 was used to classify the native barcodes. The NanoGalaxy platform (7) was used for all bioinformatic analyses. Default parameters were used, except where otherwise noted. Adapter sequences were trimmed using Porechop v0.2.4 (https://github.com/rrwick/Porechop). Filtlong v0.2.1 (https://github.com/rrwick/Filtlong) was used to filter the reads, retaining 1G bases with a minimum length, mean quality score, and window quality score of 1,000 bp, 10, and 10, respectively. Those processes yielded 66,100 reads and 16,416 bases at N50 read length. Two circular contigs, which meant a chromosome (350× coverage) and a plasmid (545× coverage), were generated via *de novo* assembly using Flye v2.9.5 (8) in nano-raw mode with five polishing iterations. Additionally, Illumina HiSeq paired-end reads (2 × 100 bp) of the DSM 19394 (=JCM 15949) genome sequence (3) were obtained from the NCBI Sequence Read Archive (ERR387483). A filtering process using fastp v0.24.0 (9) yielded 3.64M Illumina reads with 122× coverage before alignment to two circular contigs using BWA-MEM2 v2.2.1 (10). The contigs were polished twice with Illumina reads using Pilon v1.20.1 (11).

Quality checks using CheckM (12), reorientation to dnaA, and genome annotation were performed using a web-based DFAST genome annotation tool (13). The genome completeness and contamination rates were 99.35% and 0.65%, respectively. The complete genome contains a circular chromosome (2,915,962 bp, 51.71% GC content) and a circular plasmid, pJCM15949 (30,910 bp, 39.78% GC content). Annotation predicted 2,729 coding sequences (CDSs), 65 tRNA genes, and 18 rRNA genes in the chromosome.

## Data Availability

The complete genome sequence of *L. acidifarinae* type strain JCM 15949 has been deposited in DDBJ (the Nucleotide accession numbers AP040118 and AP040119) under BioProject PRJDB20195. The raw sequence data are available in the DDBJ Sequence Read Archive with the accession number DRX636836. The detailed DNA extraction protocol is shown in figshare (https://doi.org/10.6084/m9.figshare.28787192.v1).
